# Prize Essays

**Published:** 1866-10

**Authors:** J. T. Banks, J. G. Westmoreland, F. O. Dannelly, N. B. Drewry, D. C. O’Keefe

**Affiliations:** Committee on Prize Essays; Committee on Prize Essays; Committee on Prize Essays; Committee on Prize Essays; Committee on Prize Essays


### PRIZE ESSAYS.

The Committee on Prize Essays hereby invite the attention of their professional brothers to the following resolution, adopted by the Medical Association of Georgia at its last meeting:

"*Resolved*, That the sum of one hundred dollars be hereby offered by the Association for the best prize essays: $50 for the best; $30 for the second, and $20 for the third."

The essays must be written on medical or scientific subjects of interest to the profession, and sent to J. T. Banks, M. D., chairman of Committee on Prize Essays, Griffin, Ga., on or before the first day of March, 1867. Each essay must be accompanied by a sealed envelope containing the *name* and *address* of the author, and having a *sentiment* or *motto* inscribed upon it; the same sentiment or motto must also be inscribed upon the essay.

J. T. Banks, M. D.,
J. G. Westmoreland, M. D.,
F. O. Dannelly, M. D.,
N. B. Drewry, M. D.,
D. C. O'Keefe, M. D.,
*Committee on Prize Essays.*

